# Effects of Candelilla and Carnauba Wax Incorporation on the Functional Properties of Edible Sodium Caseinate Films

**DOI:** 10.3390/ijms21249349

**Published:** 2020-12-08

**Authors:** Sabina Galus, Margaux Gaouditz, Hanna Kowalska, Frédéric Debeaufort

**Affiliations:** 1 Department of Food Engineering and Process Management, Institute of Food Sciences, Warsaw University of Life Sciences-SGGW, 159c Nowoursynowska St., 02-776 Warsaw, Poland; hanna_kowalska@sggw.edu.pl; 2 IUT-Dijon-Auxerre, Département Génie Biologique, 7 Boulevard Docteur Petitjean, B.P. 17867, F-21078 Dijon CEDEX, France; margaux_gaouditz@wanadoo.fr (M.G.); frederic.debeaufort@u-bourgogne.fr (F.D.); 3 BioEngineering Department, Université de Bourgogne Franche-Comté, AgroSup Dijon, PAM UMR A 02.102, F-21000 Dijon, France

**Keywords:** edible films, sodium caseinate, candelilla wax, carnauba wax, physical properties

## Abstract

The aim of this study was to evaluate the effects of candelilla (CAN) or carnauba wax (CAR) incorporation on functional properties of edible sodium caseinate (CAS) films. Glycerol and Tween-80 were used as the plasticizer and the emulsifier, respectively. The results showed that the incorporation of waxes increased film opacity, total color differences (∆E), and mechanical resistance and reduced film lightness, water vapor permeability (WVP), and elongation at break. Scanning electron microscopy showed heterogeneous structure of emulsion films with regular distribution of lipid particles. A different internal arrangement was observed as a function of the film composition with both layered and incorporated film structure. Films containing candelilla wax exhibited more regular lipid reorganization, which resulted in better water vapor barrier efficacy and mechanical resistance in comparison to control films. The presence of Tween-80 resulted in better dispersion of lipid particles in film-forming solutions and lower water solubility, lightness, film opacity, and water vapor permeability, whereas the total color differences (∆E) were significantly larger and the improvement in mechanical properties was also achieved.

## 1. Introduction

Development of edible films for application as packaging materials is a subject of great interest due to the partial replacement of synthetic polymers. This approach may offer opportunities that would benefit both consumers and manufacturers by providing environmentally friendly packaging and less waste from the packaging industry. In addition, edible films, when used as protective coatings, can control mass transfer of substances (i.e., water vapor, oxygen, carbon dioxide, lipid, flavor, and/or aroma) with a resulting increase in quality and shelf life of food products. Edible films and coatings are traditionally used to improve the appearance and the preservation of food products [1]. On the other hand, the use of edible coatings is a promising technology to preserve the quality of whole or fresh-cut minimally processed fruits or vegetables [2]. The coating composition is chosen as a function of the desired application, and this composition is very important to determine the functional properties of the film in the product to be coated [3,4].

Various bio-based polymers have been investigated as components of edible films or coatings. Hydrocolloids, both polysaccharides and proteins, are the most popular biopolymers used in the production of edible materials. They can be obtained from such sources as plants, animals, or microorganisms [5]. Cellulose derivatives, starches, alginates, pectins, chitosans, pullulan, and carrageenans are the most common polysaccharides used in the production of edible films and coatings, whereas among proteins, the most popular are soybean proteins, wheat gluten, corn zein, sunflower proteins, gelatin, whey, casein, and keratin [6]. However, these materials are hydrophilic in nature [7]. Therefore, different types of oils and fats are incorporated into the hydrocolloid matrix in order to improve their functional properties. The most popular are waxes, triglycerides, acetylated monoglycerides, free fatty acids, and vegetable oils. Among them, waxes provide the best water vapor barrier properties among biopolymer-based films because of their high hydrophobicity, which is associated with a high content of long-chain fatty alcohols and alkanes [8]. Candelilla wax is a hard and breakable wax, insoluble in water, extracted from the wax cover of the stalks of candelillas shrubs (generally, of *Euphorbia cerifera* or *Euphorbia antisyphilitica*). It consists of hydrocarbons (approximately 50%, from C29 to C33), free fatty acids of alcohols and resins, but a relatively low quantity of volatile esters. This vegetable origin wax is classified by the Food and Drug Administration as a substance generally recognized as safe (GRAS) for food applications [9]. Carnauba wax, the most popular vegetable wax in terms of possible food applications, is obtained from leaves of the carnauba (*Copernicia prunifera* (Miller) H. E. Moore), a Brazilian palm. This wax is the hardest wax with the highest melting point among natural waxes, which is characterized by low solubility and high content of aliphatic esters and diesters of cinnamic acid [10,11].

Sodium caseinate is obtained from milk casein and is a popular ingredient in the food industry due to its good functional properties, such as solubility, emulsifying capacity, and its stability during heating. This hydrocolloid is composed of a soluble mixture of disordered hydrophilic proteins having a strong tendency to associate into small protein particles which coexist in equilibrium with the free casein molecules [12]. In general, protein-based edible materials are moisture-sensitive and this hydrophilic property is an excellent barrier to non-polar substances such as oxygen, carbon dioxide, or some aroma compounds [13], whereas it provides a poor barrier to water vapor. Therefore, the functional properties of such materials are modified by their composition. The incorporation of hydrophobic substances into protein matrixes improves their barrier properties due to the presence of discontinuities in the polymer network [14]. The physical properties of sodium caseinate-based films have been modified in order to obtain films with improved functional properties by incorporation of different lipids, including tung oil [15], linseed oil resin [16], maize germ oil bodies [17], oleic acid [18], and oleic acid/beeswax mixtures [14,18,19]. Fabra et al. [20] reported that saturated fatty acids affected the sodium caseinate film properties in a particular way due to the formation of bilayer structures which limited water vapor permeability, resulting in more opaque and less flexible films. On the other hand, the incorporation of oleic acid and beeswax was less effective, as water vapor barriers and films were more flexible without significant changes in film transparency. However, studies on adding candelilla wax and carnauba wax to sodium caseinate films are lacking. Therefore, the aim of the present study was to evaluate the effects of the combined presence of candelilla or carnauba wax and an emulsifier (Tween-80) on functionalities of edible sodium caseinate films. The effects of these additives on the particle size distribution in film-forming emulsions and microstructure of analyzed films were investigated. The hydrophilicity, water solubility, optical, barrier, and mechanical properties of sodium caseinate films were also characterized.

## 2. Results and Discussion

All the obtained sodium caseinate films with candelilla and carnauba wax were visually homogeneous, without cracks or pores. Apart from the milky color in comparison to the control film, no macroscopic change of films was observed, and waxes were completely incorporated in the matrix with smooth surface and flexible structure. All the analyzed films presented different composition (Table 1); however, a homogeneous surface on the support side (Petri dishes) and a slightly rough surface on the air side were observed for each film. The wax was rather well distributed throughout the film on either side. However, probably, more lipid globules were concentrated on the air side, which had driven the particle toward the surface with the migration of the solvent during the drying-induced changes. There were no visual differences between emulsion films obtained with and without an emulsifier (Tween-80). These observations are attributed to the relatively low amount of wax, whereas Chevalier et al. [21] observed heterogeneous and rough structure for rennet casein films with the addition of candelilla wax at 5%. Emulsification is crucial, so a low concentration of lipid particles shows a positive effect when dispersion is steady and in most cases stabilized by a biopolymer [22]. During the production and storage of protective coatings, their stability is an important parameter which can guarantee their further application on foods as well as the fabrication of films by casting or another method. In this context, functional coatings based on pectin, aloe mucilage, and candelilla wax at the concentration of 0.3% (*v*/*v*) have been shown as effective barriers to control endocarp damage by fungus invasion and protect avocados during storage [23].

### 2.1. Particle Size Analysis

**Figure 1:**
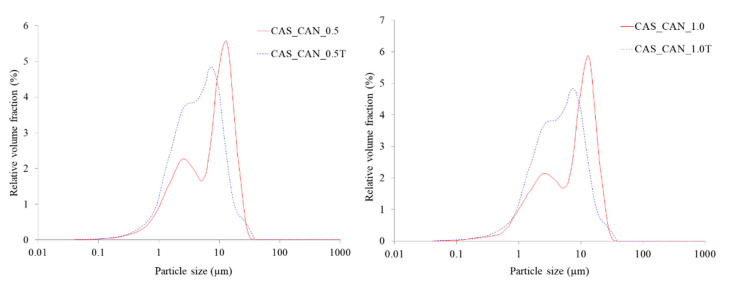
Droplet size distribution of film-forming emulsions containing candelilla wax at 0.5 and 1.0%. Abbreviations: CAS—sodium caseinate, CAN—candelilla wax, T—Tween-80.

**Figure 2:**
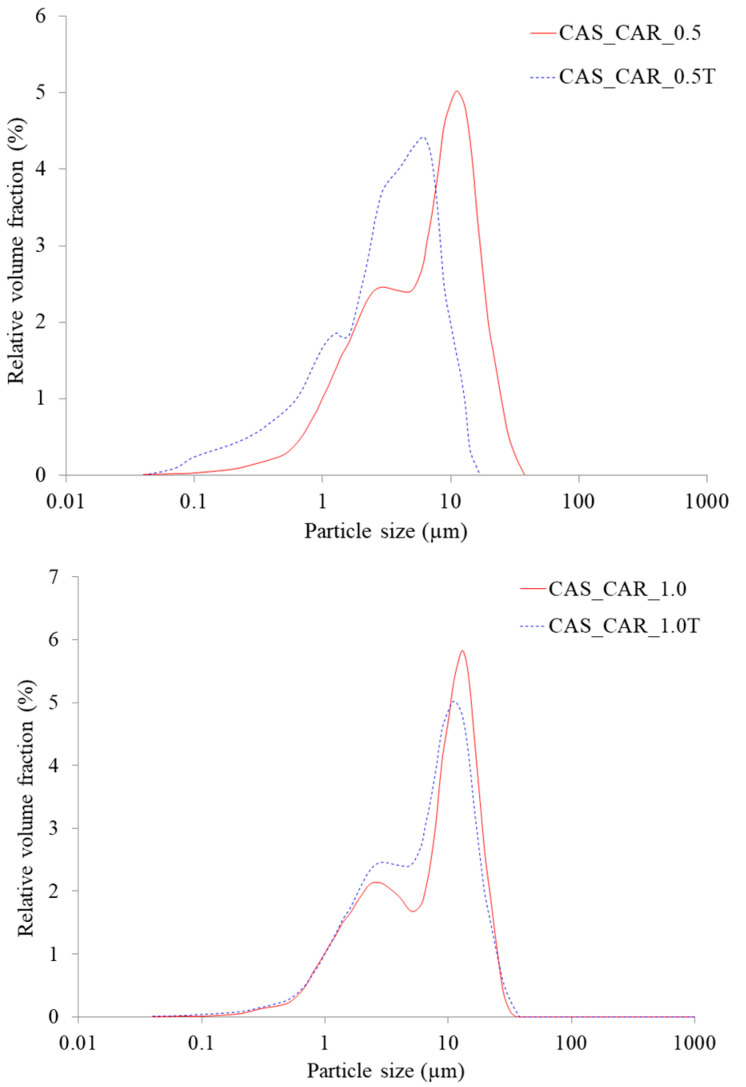
Droplet size distribution of film-forming emulsions containing carnauba wax at 0.5 and 1.0%. Abbreviations: CAS—sodium caseinate, CAR—carnauba wax, T—Tween-80.

Film-forming emulsions were visually homogeneous and the lipid droplet size distribution was measured to show the level of dispersion of wax in the film-forming matrix. A bimodal distribution for both candelilla and carnauba wax was observed and extended from 0.1 to 50 µm (Figure 1 and Figure 2). The two peaks show that the majority of lipid droplets were 2.6 and 13 µm for candelilla wax and 2.6–3.0 and between 11 and 13 µm for carnauba wax. It can be observed that the increase in the concentration of wax from 0.5 to 1.0% in film-forming solutions did not change the shape of curves for lipid distribution. However, the presence of Tween-80 showed a significant difference for both lipid particle size and their distribution. This is attributed to the hydrophobic nature of waxes, therefore, they cannot be properly dispersed in hydrophilic film-forming solutions, therefore, a negative effect on the transparency of the films was also observed. It is crucial to dissolve waxes with surfactants with a high hydrophilic/lipophilic balance (HLB) such as Tween-80 and the resulting film need to be subjected to the homogenization process [24].

A decrease in particle size in emulsions containing Tween-80 can be observed. The values ranged from 13 to 7.5 µm for both concentrations of candelilla wax and from 11 to 6.5 µm and from 13 to 11 µm for carnauba wax at 0.5 and 1.0%, respectively. This is due to the emulsifying property of the surfactant that causes a reduction in surface tension and an increase in solubility in water. In addition, a nonionic surfactant such as Tween-80 prevents protein molecules from completely coating the wax droplets. This resulted in the creation of chains and nets which provide an improved structure in the matrix and a firmer texture after drying. On the other hand, the presence of a surfactant prevents negative mechanisms that probably occurred in film-forming solutions, including lipid aggregation or coalescence. Generally, the lipid droplet distribution in aqueous solutions depends on the conditions of the homogenization process, including homogenizer type, temperature and time of the process. The lipid distribution is also connected with the melting point of waxes, which is lower for beeswax (65 °C) in comparison to candelilla and carnauba wax (85 °C). In general, the lower the melting point the easier the emulsion formation and therefore reduction in droplet size.

The lipid particle droplet size for analyzed emulsions was higher in comparison to those presented for beeswax or carnauba wax in whey protein [25] and cassava starch formulations [10] using a similar rotor/stator homogenization method. However, both authors observed the same phenomenon as presented in this study, i.e., an increase in the particle size of carnauba wax, which was from 11 to 13 µm for the second peak (Figure 2) as a result of increased concentration of wax in the film-forming solution. Similar behavior was presented by Vargas et al. [26] for chitosan films incorporated with oleic acid. Aguirre–Joya et al. [23] recently reported that particle size distribution did not detect candelilla wax in pectin film-forming emulsions containing aloe mucilage and polyphenol *Larrea* extract in the range of the size measurement limit of the equipment (9000 nm), indicating that wax particles were larger than 9 µm. Those studies have provided some evidence that the lipid distribution in biopolymer film-forming solutions is strongly affected not only by the lipid type and concentration, but also by processing conditions and compositions of the aqueous phase which can vary according to the pH, processing treatments, and the interactions between the lipid and the polymer and/or with different additives such as plasticizers and other functional substances. Generally, the values for candelilla wax were slightly higher than for carnauba wax, but only for d50 and d90 and at the concentration of 0.5% were the differences statistically significant (Table 2). However, a decrease in the lipid particle size was observed for all samples due to the presence of Tween-80, which is a surfactant of great interest because of its emulsification, stability, solubilization, and non-toxic properties [27]. The differences for d10 were not significant with the greatest reduction from 1.18 to 1.04 µm for a film with carnauba wax at 1%. However, for d50 and d90, a decrease of about 50% was observed as a result of the emulsifying property of this water-soluble agent and the presence of a lipophilic group of oleic acid. Keeping this in view, waxes with the presence of Tween-80 showed smaller lipid particle sizes and probably decreased surface tension, consequently giving a better dispersion in film-forming solutions. In general, it is observed that relatively low amounts of Tween-80 are often used as an effective emulsifying agent for other edible lipid-containing protein-based films including whey [28], surimi [29], and myofibrillar proteins [30].

### 2.2. Film Microstructure

**Figure 3:**
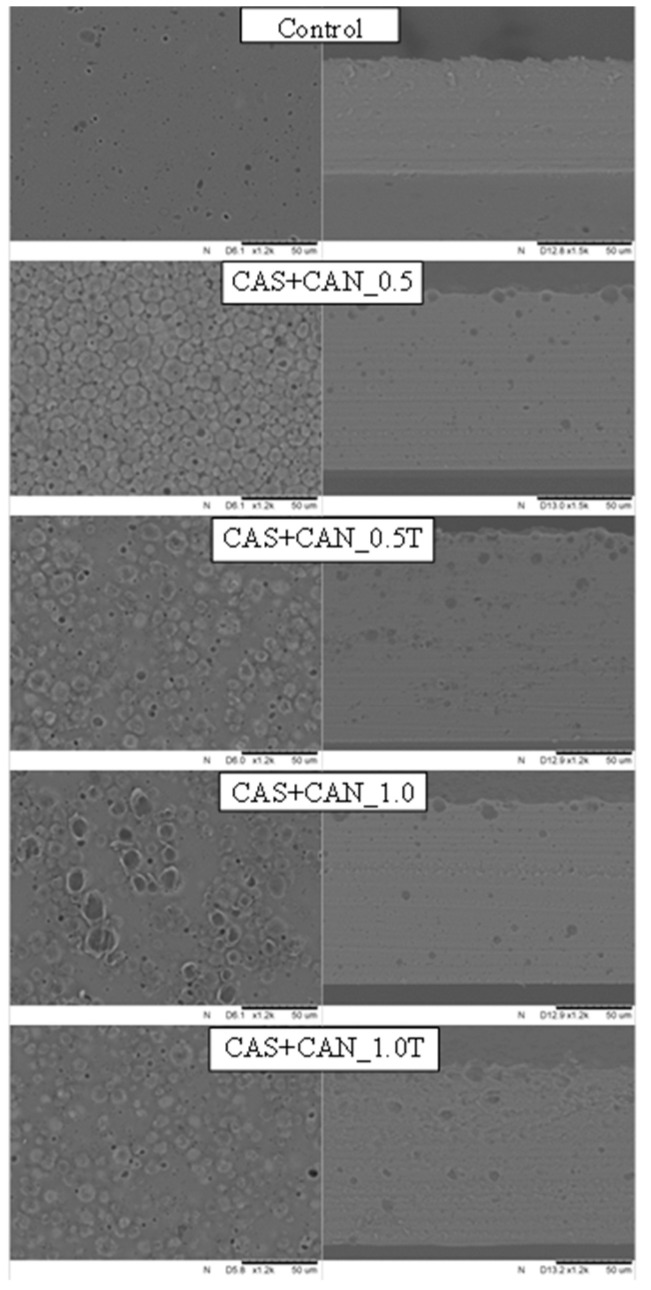
Scanning electron micrographs of surfaces (×500) and cross-sections (×1500) of sodium caseinate films without and with candelilla wax at 0.5 and 1.0%. Abbreviations: CAS—sodium caseinate, CAN—candelilla wax, T—Tween-80.

**Figure 4:**
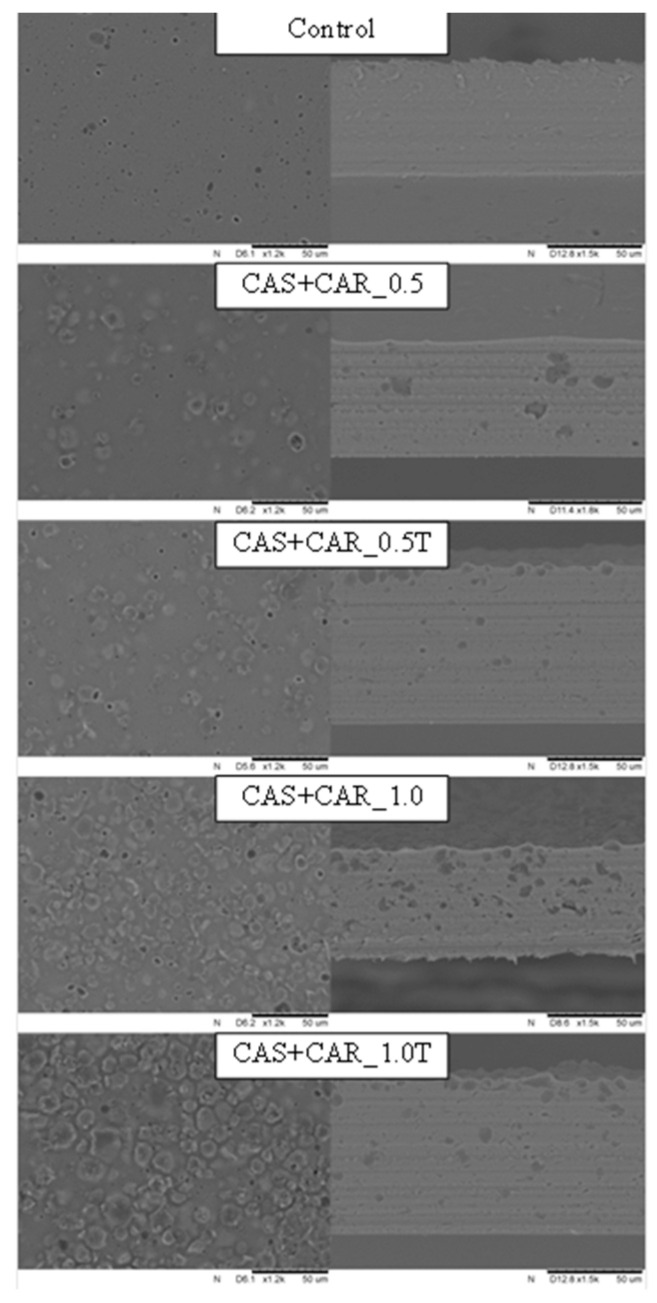
Scanning electron micrographs of surfaces (×1500) and cross-sections (×1800) of sodium caseinate films without and with carnauba wax at 0.5 and 1.0%. Abbreviations: CAS—sodium caseinate, CAR—carnauba wax, T—Tween-80.

The microstructure was analyzed qualitatively in order to verify the features promoted by different concentrations of candelilla and carnauba wax and presence or absence of Tween-80. Figure 3 and Figure 4 show scanning electron micrographs of the film surface and cross-sections of control films and the sodium caseinate films containing candelilla and carnauba wax. It can be noted that different internal arrangements are observed as a function of film composition. While the control film containing only protein and glycerol showed a smooth and homogeneous microstructure, the films prepared with the addition of wax showed structural discontinuities due to the lack of miscibility of components. The irregular shape of wax particles in films reveals the solid state of these particles during film formation. Therefore, these observations agree with the wax particle size distribution and film microstructure observed by scanning electron microscopy. Smaller wax droplets were observed in films as a result of the addition of Tween-80 (Figure 3 and Figure 4). Aggregation and coalescence of lipid droplets during film drying probably occurred, since different lipid droplet distributions in the matrix with irregular lipid globules are present in the protein network on the evaporation surface. Small droplets probably migrated toward the evaporation surface during casting and the beginning of drying, before the viscosity increased significantly. This flotation phenomena can explain aggregation and coalescence because of a very high concentration of lipid particles at the surface of solvent evaporation. The intensity of such phenomena depends on the concentration of the lipid, the particle size in the initial film-forming emulsion, the viscosity of the continuous phase, and the properties of the interfacial surface of wax droplets.

Formation of lipid droplets and their development during film drying are expected to lead to interruption of the protein matrix, increasing the internal heterogeneity and the surface roughness of the film [31]. The increase in surface roughness is principally due to the different mechanisms that can occur in emulsions, such as creaming, aggregation, or coalescence, as well as during film drying. Keeping this in mind, the irregular aspect of the film surface can be observed for wax-containing films, and these irregularities were more accentuated as wax content increased. This is in agreement with the higher ratio of the non-polar lipid with a higher melting point and probably greater droplet size, which might imply that the coalescence and creaming may occur on a greater scale. According to Jiménez et al. [32], solvent evaporation causes changes in components’ concentrations and in the emulsion liquid phase viscosity, leading to lipids’ aggregation and creaming, thus affecting the inner structure and the film surface and therefore affecting other physical properties of edible films. In addition, wax addition promotes discontinuities in the polymer matrix of the dried film, modifying its mechanical resistance. In general, unlike the control film, wax-containing films showed discontinuity in the matrix, with a rather irregular surface, with the presence of pores and lipid globules’ agglomeration. This rough surface was created by aggregation of wax droplets and subsequent recrystallization during film drying. Therefore, the presence of wax crystals on the surface of the caseinate film matrix resulted in an increase in the surface roughness. Similar observations have been noted previously for biopolymer films obtained with the addition of waxes [33,34,35]. However, the cross-section of the films showed a more cohesive structure, with closed polymeric networks and fewer pores, but with wax clusters. The polymer structure was less cohesive than the control formulation matrix, possibly due to low glycerol concentration and high wax percentage, providing more rigid films. Candelilla wax is composed of approximately 50% hydrocarbons, whereas carnauba wax contains 12–20% cinnamic and hydroxyl-cinnamic acid esters [33], and these particular esters are known for their unique hydrophobicity effect, which dominated the lipid droplet distribution and hydrophobic nature of sodium caseinate films. Thus, films containing candelilla wax exhibited more regular lipid reorganization, which led to better water vapor barrier efficacy and mechanical resistance. The harder structure of films with carnauba wax can be explained by a higher melting point [25], which might cause difficulty in the production of stable and uniform emulsions. The results correlate with the mechanical properties of sodium caseinate films containing candelilla wax, which were more flexible that those obtained with carnauba wax. In addition, this property also resulted in better water vapor barrier resistance of the analyzed films.

The cross-sections of the film microstructure may explain the results obtained for mechanical properties and barrier films’ analyses. The cohesive polymer network promoted effective gas and moisture barriers, and the presence of wax clusters reduced the mechanical strength and flexibility of the films due to the formation of brittle points in the polymer matrix. In general, when the emulsifier (Tween-80) was added, the dried films had a smooth surface, and only negligible cracking was observed. In general, the hydrophobic character of waxes is attributable to their chemical composition and the orientation of their molecules. The concentration of each constituent shows different hydrophobic character of the wax, thus resulting in different physical properties of wax-containing films. As can be observed from the surface and cross-sections of films with wax, lipid globules were located in a higher amount on the top of the film, suggesting that sodium caseinate/wax films prepared by emulsification have two forms, layered and incorporated. This is probably due to the wax melting points, which make them more unstable, thus promoting progression of the destabilization mechanism during film drying and greater accumulation of lipid aggregates on their surface. These observations indicate only that more research is needed in order to unify the film structure and obtain uniform and compact materials.

### 2.3. Water Solubility

Water solubility determination gives information about film resistance when in contact with wet foods. None of the analyzed films lost their integrity or dissolved after 24 h of storage in water, which might be an indicator of a highly stable protein network. This stable protein network was obtained as a result of the denaturation process during film-forming solution preparation. In addition, water solubility analysis was done after drying (105 °C for 24 h), and thus a more compact structure was obtained due to the drying process. Preliminary studies showed that for the swelling in water analysis, which does not require drying, all the samples demonstrated excellent ability to absorb water, resulting in disintegration and impossibility to measure their swelling capacity. Similar observations had been reported previously for soy protein isolate films [36]. This behavior may also be attributed to the water retention capacity of hydrocolloid matrix as a hydrophilic substance. Solubility in water of sodium caseinate films incorporated with candelilla and carnauba is shown in Table 3. The highest solubility was exhibited for the control film, 45.53%. The results ranged from 43.41 to 43.92% and from 44.46 to 45.42% for candelilla- and carnauba-containing films, respectively. The addition of waxes to caseinate films results in a significant reduction in water solubility, except for the film containing carnauba wax at 0.5%. Lower solubility of lipid-containing films in comparison to control films is a consequence of the hydrophobic character of the waxes. In addition, water solubility decreased with higher content of wax, and there is a significant difference between the candelilla wax and the carnauba wax.

It can also be observed that the presence of Tween-80 slightly increased water solubility of films due to its hydrophilic character, although those differences were not statistically significant. Several research groups have reported a decrease in water solubility of biopolymer films as a result of wax addition. Reduction in water solubility due to the incorporation of beeswax or candelilla wax was noted by Kim and Ustunol [37] and Soazo et al. [38]. The authors connected the results of constant total solid values in the formulation with the reduction in the soluble matter in the films due to the incorporation of wax, and, consequently, the lowered water solubility. Dos Santos et al. [39] noted a 60% reduction in water solubility of chitosan films when carnauba wax was added at 15%, but higher amounts of wax addition showed increased values, which was probably due to the higher concentration of Tween-20 and its hydrophilic effect. A decrease in water solubility as a result of wax addition was also observed for fish skin gelatin films incorporated with beeswax and carnauba wax [35], whey protein isolate/beeswax films [40,41], and cassava starch/carnauba wax films [34,42].

In general, water solubility at the range of those presented in this study is similar to other protein-based films [43] and the values are suitable for application to perishable foods, such as fresh-cut fruits or vegetables [34]. Matta et al. [44] reported that addition of plasticizer, in particular of glycerol, has a great influence on the solubility of starch films, due to its hydrophilic character. Glycerol is a very hygroscopic substance, which interacts with the film matrix by increasing the space between the polymer chains, facilitates water diffusion and, consequently, increases film solubility in water.

### 2.4. Film Opacity

Opacity is a crucial parameter for edible films in order to use them as a protective coating for food. It is well known that those materials should be transparent or only slightly opaque to limit the loss of light-sensitive compounds such as vitamins or minerals. Nevertheless, when these structures are too opaque, the product will not seem any more natural. The opacity of the analyzed films significantly increased from 2.28 to 10.55 A·mm^−1^ as a function of wax concentrations (Table 4), which may impair the applicability of emulsion films when high transparency is required. It was noted that the films prepared with carnauba wax were more opaque than the films prepared with candelilla wax; however, those differences were not statistically significant.

Carnauba wax possesses a majority of esters of fatty acids which, in an aqueous environment, probably become organized in a two-dimensional network. Thus, the presence of Tween-80 resulted in a decrease in the opacity which is attributed to the smaller particle size of wax (Figure 1 and Figure 2). Bearing this in mind, better organization of lipid particles in the sodium caseinate film matrix showed an effective barrier against a visible spectrum of the UV–VIS light (600 nm), which may be a critical factor for practical application of the analyzed materials as an edible coating for specified food products. Different structural rearrangement occurs during the emulsified film drying process which may cause several mechanisms such as creaming, aggregation, or coalescence, which affect film homogeneity and visible light scattering through the films [39], thus resulting in limited film transparency. Numerous studies have previously reported a decrease in film opacity or transparency of biopolymer films as a consequence of addition of waxes [19,33,35,39,45,46,47].

### 2.5. Color

The color of edible films is important considering the practical applications such as packaging material or edible coating for the food industry [48]. The L*, a*, and b* color parameters and total color difference (ΔE) values of sodium caseinate films as a function of wax concentration and presence of Tween-80 are presented in Table 4. The highest lightness (parameter L*) was obtained for the control film (98.9), whereas all wax-containing films were significantly darker, with L* values from 94.1 to 97.2. It can be observed that the films containing carnauba wax were darker in comparison to the candelilla ones. However, significant differences are observed only between the films containing wax at 0.5 and 1.0%. This can be attributed to the color of the wax itself as well as the miscibility and integrity with the polymer in the continuous polymer matrix. The presence of Tween-80 led to the lowered lightness, which was significant for the film containing candelilla wax at 0.5% and for carnauba wax at 1.0%. This is connected with the smaller droplet sizes as well as their better and regular distribution in film-forming emulsions before casting, which was probably maintained during film drying.

In general, sodium caseinate forms transparent films with a light milky color which provide high lightness [15]. It is well-known that lipid-containing films show a tendency to reduced film transparency [36]. A decrease in lightness as a result of the addition of carnauba wax was also observed for chitosan films [39]. A similar tendency was reported by Zhang, Simpson, and Dumont [35] for the gelatin films incorporated with beeswax.

All the films showed negative a* parameter values which means that films had high greenness. Significant differences were observed for all wax-containing films in the range from −3.22 to −2.21 in comparison to the control one (−0.85). Both the presence of Tween-80 and the increasing amount of wax concentration from 0.5% to 1.0% showed a tendency to the reduction in the value of parameter a*. Carnauba wax addition gave higher greenness than candelilla wax. There was an increase in yellowness with increasing wax concentration, based on the increase in the positive b* parameter. The presence of Tween-80 led to higher values in parameter a*, but only the values for films at the 0.5% concentration of candelilla wax were statistically significant. It can be observed that carnauba wax gave a greater yellowness than candelilla wax. The total color difference (∆E) significantly increased from 1.73 for the control film to 12.3 for the film containing Tween-80 and carnauba wax at 1.0%. With higher concentration of wax, greater total color differences (∆E) were observed. The values were lower for candelilla-containing films (2.75–4.75) and higher for those with carnauba wax (7.07–10.9). The addition of the emulsifier Tween-80 resulted in an increase in total color differences (∆E), but the differences were statistically different only for the films containing candelilla wax at the concentration of 0.5%. Similar observations have been reported by Muscat et al. [33] for high-amylose starch films with the addition of beeswax, candelilla and carnauba wax. In general, several research groups have described the widely known and scientifically proven phenomenon that the addition of different lipid components contributes to intensification of the color of biopolymer films. This is also dependent on their type and concentration [8,15,45]. The analyzed films were characterized by values of total color difference higher than 3, which indicates that they were noticeable. Thus, incorporation of candelilla and carnauba waxes will visually affect the appearance of the coated food product.

### 2.6. Water Vapor Permeability

The results of water vapor permeability of sodium caseinate films incorporated with candelilla and carnauba wax are presented in Table 5. As can be seen from this table, the highest value, 4.75 × 10^−10^ g·m^−1^·Pa^−1^·s^−1^, was obtained for a film without wax addition. The inclusion of waxes in the films significantly lowered water vapor permeability of sodium caseinate films due to the increased hydrophobicity as a consequence of presence of the lipid phase. However, water vapor permeability values for lipid-containing films are usually higher in comparison to the monolayer of wax films, since a continuous wax layer is more effective than heterogeneous polymer-based films obtained with the addition of waxes. Thus, moisture transfer occurs through the continuous phase for the film that is a protein layer rather than lipids. No significant differences were observed for films with different wax concentrations, indicating that the levels of 0.5% and 1.0% were not efficient in changing water barrier efficiency of the analyzed films. The significant reduction in water vapor permeability of high-amylose starch films with 5 and 10% candelilla and carnauba wax was observed by Muscat et al. [33]. On the other hand, Zhang, Simpson, and Dumont [35] noted a significant decrease in water vapor permeability of fish skin gelatin films when carnauba wax was used at the concentration of 0.5% and 0.75%. However, the improvement in water vapor barrier properties may be due to the presence of a rough surface in the analyzed films, as can be seen in the scanning electron micrographs in Figure 3 and Figure 4. These micrographs show the presence of both wax droplets and the subsequent recrystallization during film drying. It is well-known that the main reason for incorporation of lipids into biopolymer films is to improve their water barrier property, which is usually low, since most of the edible films are hydrophilic [22]. Therefore, several research groups have reported a decrease in water vapor permeability of edible films as a consequence of wax addition, including whey protein isolate [49], soy protein isolate [45], pea protein isolate [46], fish skin gelatin [35], chitosan [39], konjac glucomannan [47], cassava [34,42], and high-amylose starch-based films [33]. However, Brzoska et al. [18] recently reported an increase in water vapor permeability of sodium caseinate films prepared with the addition of beeswax at the concentration of 1.0% without the presence of a plasticizer. A similar tendency was presented by Kowalczyk and Baraniak [45] for pork gelatin/candelilla wax films. The authors showed that the homogeneous distribution did not present a successful barrier to water transfer. Therefore, water vapor most likely still easily penetrated between lipid globules dispersed inside a continuous protein network. Those observations only confirm the already known phenomenon that gas and water vapor barrier properties of edible films and coatings vary remarkably. In addition, those differences depend not only on the film composition (different film-forming biopolymer or plasticizer type and concentration), but also on the formation method or modification treatment, as well as the methodologies used for determining water vapor permeability. Moreover, the microscopic structure has to be taken into account as dispersed wax particles or a multilayer structure, which impact the barrier properties more than other parameters considered.

The addition of an emulsifier decreased the water vapor permeability values, but statistically significant differences were observed only for the film containing 0.5% candelilla wax. Nevertheless, this tendency agrees with the observations for droplet size distribution, indicating that the emulsifying property affected the intermolecular interactions between molecules of water and waxes and less water could migrate through the caseinate films. In addition, this statement is in line with the results of the film solubility in water when no significant difference was observed between films with and without the emulsifier. Nevertheless, many aspects such as wax concentration or biopolymer used are crucial for gas barrier properties. Muscat et al. [33] reported that high-amylose starch films incorporated with different waxes at the concentration of 5 and 10% showed higher water vapor permeability values for films with Tween-80 in comparison to control films and films without an emulsifier. Therefore, the mechanisms of action of Tween-80 as an emulsifier vary for different polymer matrixes. Besides the fact that the emulsifier complements the plasticizing behavior, being present between the sodium caseinate molecules probably promoted formation of the protein–protein binding. This could explain the more compact structure for wax-containing films, which showed structural integrity by decreasing the free volume between the protein chains, thus reducing diffusion of water vapor through the films. These observations only show the complex phenomenon of film barrier properties. Furthermore, Tween-80 is a hydrophilic emulsifier which should in theory increase water vapor permeability, but it seems to be dependent on the biopolymer type and the intermolecular interactions in film-forming solutions or during film drying. In addition, emulsifiers improve adhesion of the film, which probably has an impact on water barrier efficiency. Therefore, by introducing wax together with an emulsifier in the sodium caseinate films, it was possible to produce films with improved water vapor barrier properties.

### 2.7. Mechanical Properties

Mechanical resistance is an important property for edible films and coatings due to being related to the film and coating durability as well as to the ability of coatings to enhance mechanical integrity of coated foods. The tensile strength, elongation at break, and Young’s modulus of sodium caseinate films obtained with the addition of candelilla and carnauba wax are presented in Table 6. The tensile strength is the maximum tensile stress that a material can sustain and is taken to be the maximum force exerted on the test specimen during the test divided by the original cross-section of the specimen. It can be observed that the addition of waxes showed higher values from 1.91 to 2.60 MPa in comparison with the control film (1.65 MPa) and a tendency to greater values with increased wax concentrations, although these differences were not statistically significant. Usually, the addition of lipids results in the weaker mechanical resistance. However, regarding the opposite effect for wax-containing films, it is probably attributed to the presence of wax globules and their distribution in the film matrix. In addition, molecular chains formed in a composite structure led to a more compact structure which resulted in better mechanical resistance. Nevertheless, the presented values for tensile strength are rather low and similar to those for plant or animal protein films obtained by others [18,35,45,49].

Regarding the type of wax used, there is no difference, indicating that both lipid sources showed similar function in the small amounts of analyzed films. Muscat et al. [33] reported a slight decrease in tensile strength of high-amylose starch films as a result of beeswax, candelilla and carnauba wax addition. Similar observations were noted for carboxymethyl cellulose/candelilla films [45] and cassava/carnauba wax films [34,42]. The addition of the emulsifier to the analyzed films caused an increase in tensile strength, which was greater and significant only for candelilla-containing films. This could be explained by the better lipid distribution in film-forming emulsions before drying (Figure 1), the nature of the wax, and the interaction between protein, wax, plasticizer, emulsifier, and water molecules, which affected the formation of the film structure and their compatibility. However, these observations are contrary to the results presented for high-amylose starch films, where the addition of Tween-80 to films containing beeswax at the concentration of 5% caused a largely (approx. 50%) decrease in tensile strength [33]. The authors stated that the emulsifier interfered with the interaction of starch with wax probably by occupying the space between starch molecules. Nevertheless, these interactions strongly depend on the biopolymer and the lipid used in the formulation. In addition, the presence of a plasticizer, such as glycerol used in this study, affects mechanical resistance. Al-Hassan and Norziah [50] reported that protein-based edible films plasticized with the addition of glycerol had lower values of tensile strength, indicating that films were more flexible due to the plasticizing effect, which increased the mobility of the polymer chains. The flexibility increases as a function of plasticizer content, and this is related to the structural changes in the sodium caseinate network due to less dense films and greater movements of the component molecules in the biopolymer matrix.

The elongation at break as the maximum tensile strain that a material can sustain measures the film’s ability to stretch and is expressed as the ratio between the sample’s length at break and its original length. As can be observed from Table 6, the addition of waxes led to lower elongation at break, from 142.0% to 63.7%. An increasing amount of both waxes used resulted in the reduction in film elasticity, which was also observed for protein films incorporated with candelilla wax [45] and beeswax or carnauba wax [35]. Lipid particles induce discontinuities that may reduce the elongation at break because of preferential breaking zones. Liquid fraction could play a role as a lubricant by increasing tensile strength and elongation at break, whereas Tween-80 could act as a plasticizer resulting in higher elongation and lower mechanical resistance. However, this tendency must be an amount-dependent factor, since different results were presented by other research groups. An increase in elongation at break was noted for beeswax/sodium caseinate films [18], high-amylose starch films as a result of candelilla and carnauba wax addition [33] and for carnauba wax/cassava starch films [42]. However, Janjarasskul et al. [49] reported no change in elongation at break of whey protein films when candelilla wax was used at the concentrations of 5 and 7.5%. Moreover, the increase in concentration of candelilla wax from 0.5% to 1.5% showed a decrease in elongation at break of pork gelatin films, whereas 2% wax resulted in a significantly greater value. These observations only suggest that many factors have to be taken into account in order to provide desirable film flexibility. In addition, elongation at break strongly depends on the plasticizer concentration [51]. Candelilla and carnauba wax showed an anti-plasticizing effect on sodium caseinate films. However, the addition of Tween-80 significantly increased elongation at break, from 68% to 102.4% and from 63.7% to 114.5% for candelilla and carnauba wax, respectively. The higher the wax concentration, the lower the elongation at break and the emulsifying effect. It is due to the hydrophobic character of the wax and its interactions with the emulsifier, indicating that the presence of Tween-80 exerts a further plasticizing effect and strengthened plasticizing efficacy of glycerol. In other words, the addition of Tween-80 has an effect as if a larger amount of plasticizer is used. This is also due to the fact that the molecular size of Tween-80 is relatively small, and it could occupy the space between sodium caseinate chains, like glycerol, further increasing chain mobility and enhancing the plasticizing effect. The hydrophilic part of Tween-80 interacts with glycerol or water, facilitating its presence between sodium caseinate chains, which confirms the observation of particle size distribution, and thus film microstructure. Furthermore, natural waxes possess variable mechanical properties generally governed by their chemical composition and physical properties [22].

Young’s modulus is the ratio between the stress and deformation of a sample, calculated by the slope of the stress/strain curve obtained in an initial tensile test. As shown in Table 6, Young’s modulus for all wax-containing films was higher in comparison to the control film, but the differences were significant only for films with candelilla wax and Tween-80 and those prepared with carnauba wax with or without the emulsifier. These data showed that the addition of natural waxes to sodium caseinate films seems to have an effect on the increased Young’s modulus, even though the films containing wax maintained a greater level of tensile strength. Therefore, the synergetic effect of protein and wax molecules and/or emulsifier led to improved mechanical resistance. In summary, wax addition increased tensile strength and Young’s modulus while decreasing film elasticity and flexibility. These effects can be attributed to the development of discontinuities in the polymer network induced by wax addition observed in film microstructure (Figure 3 and Figure 4).

## 3. Materials and Methods

### 3.1. Materials

Sodium caseinate (min. ~90 g per 100 g of proteins) was purchased from Polsero Sp. z o.o. (Sokołów Podlaski, Poland). Candelilla wax and carnauba wax were obtained from Strahl & Pitsch Inc. (West Babylon, NY, USA). Anhydrous glycerol was purchased from Avantor Performance Materials Poland S.A. (Gliwice, Poland) and was used as a plasticizer in order to improve the mechanical properties of films. Food grade Tween-80 as an amphiphilic substance was obtained from Sigma–Aldrich (Saint Louis, MO, USA) and was used as an emulsifier in order to reduce surface tension of the film-forming solutions containing waxes and to improve wettability of the films. Waxes were dispersed into water film-forming solutions with the help of Tween-80. Films obtained without waxes and the emulsifier were used as the control.

### 3.2. Preparation of Film-Forming Emulsions

Aqueous film-forming solutions were prepared by slowly dissolving sodium caseinate at 8% (*w*/*w*) in distilled water under magnetic stirring using magnetic plate model RTC basic IKAMAG (IKA, Staufen, Germany) for 10 min. According to the preliminary study, glycerol at 50% (*w*/*w*) of the protein (sodium caseinate) was used as a plasticizer and Tween-80 at 0.09% and 0.18% as an emulsifier. Subsequently, the solutions were heated on a magnetic plate to 80 ± 1 °C and candelilla or carnauba waxes were added at 0.5 or 1.0% (*w*/*w*) and after melting the mixtures were homogenized at 13,500 rpm with an Ultra Turrax homogenizer model Yellowline DI25 basic (IKA, Staufen, Germany) for 3 min to obtain film-forming emulsions. The compositions of film-forming formulations are presented in Table 1.

### 3.3. Particle Size Analysis

Film-forming solution structure was determined by laser light scattering granulometry using a Cilas 1190 particle size analyzer (Orléans, France) at room temperature. Reproducibility was tested by carrying out six measurements of each replicate with the detection range between 0.04 and 2500 µm.

### 3.4. Film Formation Method

Film-forming solutions were poured onto a series of Petri dishes. To control film thickness, the same quantity of each suspension was poured onto a plate. The film-forming suspensions were dried for 24 h at 25 ± 1 °C and 50 ± 1% relative humidity (RH) in a ventilated chamber model KBF 720 Binder (Tuttlingen, Germany). During drying, the suspensions formed easily detachable films with the final film thickness of 80 ± 5 µm. Then, films were peeled off and conditioned again at 50 ± 1% RH and 25 ± 1 °C for 48 h prior to testing.

### 3.5. Film Thickness

Film thickness was measured with an electronic gauge (Metrison, Mościska, Poland) with the precision of 1 µm. Prior to film thickness measurements the electronic gauge was calibrated at 74 and 139 µm using standard sheets. Thickness of each film was measured in five places, one in the central part of the film and four around its perimeter, and the mean value was used in the calculations.

### 3.6. Water Solubility

The films were cut into 25 × 25 mm pieces, weighed, and dried at 105 °C for 24 h. After drying, films were cooled down and weighed. Then, films were individually placed in 50 mL beakers filled with 20 mL of distilled water, covered, and stored at 25 ± 1 °C for 24 h occasionally shaken. Film pieces were taken out and dried again at 105 °C for 24 h to determine the final weight of the dry matter. The measurements were done in five repetitions and the calculations from the initial and final dry weight of films were made according to the method described by Rhim et al. [52].

### 3.7. Color

The color of films was determined in 10 repetitions with a colorimeter, CR-300 (Konica Minolta, Tokyo, Japan), using the CIELAB color parameters: L* from black (0) to white (100); a* from green (−) to red (+); and b* from blue (−) to yellow (+). The color of films was expressed as the total color difference (ΔE) according to the following equation [53]:

(1) $$\Delta E=\sqrt{{\left(\Delta L*\right)}^{2}+{\left(\Delta a*\right)}^{2}+{\left(\Delta b*\right)}^{2}}$$

where: ΔL*, Δa*, and Δb* are the differentials between the color parameter of the white standard used as the film background (L* = 98.07, a* = −1.78, b* = 2.27) and the sample color parameters.

### 3.8. Film Opacity

Films were cut in 50 × 10 mm pieces and the thickness of each piece was measured in triplicate. The opacity was measured with a HEλIOS UV–VIS spectrometer (Thermo Electron Corporation, Waltham, MA, USA) with a wavelength of 600 nm. The measurements were repeated ten times for each type of film.

### 3.9. Water Vapor Permeability

The water vapor permeability (WVP) of analyzed films was determined gravimetrically using a modified ASTM E96-80 standard method, adapted to edible materials by Debeaufort, Martin–Polo, and Voilley [54] using the RH differentials of 50–100% and the temperature of 25 ± 1 °C. Film samples were placed between two rubber-based rings on the top of glass cells containing distilled water and were stored at 25 ± 1 °C and 50 ± 1% RH in a ventilated chamber model KBF 720 Binder (Tuttlingen, Germany). The water vapor permeability was measured in triplicate and calculated from the change in the cell weight versus time at the steady state using the following equation:

(2) $$\mathrm{WVP}=\frac{\Delta m\cdot e}{A\cdot \Delta t\cdot \Delta p}$$

where Δm/Δt is the weight of moisture loss per unit of time (g·s^−1^), A is the film area exposed to the moisture transfer (8.04∙10^−4^ m^2^), e is the film thickness (m), and Δp is the water vapor pressure differential between the two sides of the film (1583.5 Pa).

### 3.10. Mechanical Properties

The tensile strength (TS), Young’s modulus (YM), and elongation at break (E) of the film pieces 25 × 10 mm in size were determined using a texture analyzer, TA-XT2i (Stable Microsystems, Haslemere, UK) according to the ASTM standard method D882 [51]. The film specimens were mounted in the self-tightening roller grips of the testing machine and stretched at the rate of 1 mm·s^−1^ until breaking. The initial distance of separation was adjusted to 50 mm. At least ten replicates of each film formulation were tested. Young’s modulus (YM) was evaluated as the slope of the initial linear portion of tensile stress (*σ*) and the strain (*ε*) curves:

(3) $$\mathrm{YM}=\frac{\delta }{\varepsilon }$$

The tensile strength (TS) and elongation at break (E) were calculated according to the following equations [55]:

(4) $$\mathrm{TS}=\frac{N}{mm^{2}}$$

where N is the maximum force at rupture of the film, mm^2^ is the initial cross-sectional area of the films.

(5) $$E=\frac{D_{f}-D_{i}}{D_{i}}\cdot 100\%$$

where *D_f_* is the distance of the elongation at break (mm) and *D_i_* is the initial distance between the grips.

### 3.11. Film Microstructure

The film microstructure was observed under a scanning electron microscope, TM3000 tabletop microscope (HITACHI High-Technologies Europe GmbH, Krefeld, Germany). A 5 × 5 mm piece of film was fixed on the support using carbon paste at an angle of 90° to the surface, which allowed observation of the cross-section of the film cut with a scalpel. No particular film preparation was necessary. The films were observed at a magnification of ×1500 (surfaces) and ×1800 (cross-sections).

### 3.12. Statistical Analysis

Statistical analysis was performed using Statistica 10.0 (StatSoft Inc., Tulsa, OK, USA). The analysis of variance (ANOVA) was performed with the post-hoc Tukey’s test to detect significant differences in the film’s properties. The significance level used was 0.05.

## 4. Conclusions

In this study, the effects of candelilla or carnauba wax incorporation on properties of edible sodium caseinate-based films were investigated. Both waxes showed similar bimodal lipid droplet distribution in film-forming solutions with better dispersion of lipid particles when an emulsifier was used. Water solubility decreased when the wax content was higher, especially for candelilla wax- and Tween-80-containing films. Film opacity increased with the incorporation of wax, and the presence of an emulsifier decreased the opacity, because it fits between networks created by the wax, making the films less dense. The total color differences of the films were significantly affected by the addition of waxes, as these films exhibited higher yellow–blue coordinates and lower lightness. The candelilla and carnauba waxes showed a greater decrease in the water vapor permeability values of sodium caseinate films due to the highly hydrophobic nature of the waxes. The incorporation of the candelilla and carnauba wax caused a significant decrease in the elongation at break of the analyzed films and increased the tensile strength and Young’s modulus, indicating that wax addition provided more resistant films. The presence of Tween-80 resulted in lower water solubility, lightness, film opacity, and water vapor permeability, whereas the total color differences (∆E), tensile strength, and Young’s modulus were significantly higher. Thus, incorporation of wax together with an emulsifier in sodium caseinate films showed improved mechanical and water vapor barrier properties of sodium caseinate films. Moreover, glycerol and the emulsifier showed their plasticizing effect by the ability to reduce intermolecular hydrogen bonding, while the increase in intermolecular spacing and modified mechanical resistance of the films were observed. Water vapor permeability and water solubility were reduced by candelilla and carnauba wax, indicating that the presence of these waxes may be interesting for applications which require properties of a good water barrier and resistance. In addition, candelilla wax exhibited more regular lipid reorganization, which resulted in greater water vapor barrier efficiency and mechanical resistance. Moreover, candelilla and carnauba wax sodium caseinate films can be applied as protective edible coatings for different food products, mainly for those naturally containing lipids or those which require limitation of water vapor permeability. However, more research is needed to adapt food applications.

## Figures and Tables

**Table 1:** Composition of sodium caseinate films with candelilla and carnauba wax.

Film	CAS (%)	GLY (%)	CAN (%)	CA (%)	T (%)
Control	8	4	-	-	-
CAS_CAN_0.5	8	4	0.5	-	-
CAS_CAN_0.5T	8	4	0.5	-	0.09
CAS_CAN_1	8	4	1	-	-
CAS_CAN_1T	8	4	1	-	0.18
CAS_CAR_0.5	8	4	-	0.5	-
CAS_CAR_0.5T	8	4	-	0.5	0.09
CAS_CAR_1	8	4	-	1	-
CAS_CAR_1T	8	4	-	1	0.18

Abbreviations: CAS—sodium caseinate, GLY—glycerol, CAN—candelilla wax, CAR—carnauba wax, T—Tween-80.

**Table 2:** Particle size distribution (d10, d50, and d90) in film-forming emulsions.

Film	d10 (µm)	d50 (µm)	d90 (µm)
CAS_CAN_0.5	1.20 ± 0.13 ^a^	7.74 ± 0.09 ^d^	16.5 ± 0.05 ^e^
CAS_CAN_0.5T	1.14 ± 0.08 ^a^	4.31 ± 0.03 ^b^	11.7 ± 0.06 ^c^
CAS_CAN_1	1.24 ± 0.12 ^a^	8.04 ± 0.07 ^e^	16.7 ± 0.08 ^e^
CAS_CAN_1T	1.14 ± 0.08 ^a^	4.32 ± 0.01 ^b^	11.7 ± 0.06 ^c^
CAS_CAR_0.5	1.16 ± 0.12 ^a^	6.43 ± 0.10 ^c^	15.6 ± 0.18 ^d^
CAS_CAR_0.5T	1.08 ± 0.16 ^a^	3.08 ± 0.06 ^a^	8.17 ± 0.06 ^a^
CAS_CAR_1	1.18 ± 0.14 ^a^	7.97 ± 0.17 ^e^	16.7 ± 0.08 ^e^
CAS_CAR_1T	1.04 ± 0.11 ^a^	3.12 ± 0.03 ^a^	8.56 ± 0.08 ^b^

Mean values ± standard deviations. Different superscripts (^a–e^) within any column indicate significant differences between samples at *p* < 0.05. CAS—sodium caseinate, CAN—candelilla wax, CAR—carnauba wax, T—Tween-80.

**Table 3:** Water solubility of sodium caseinate films with candelilla and carnauba wax.

Film	Water Solubility (%)
Control	45.53 ± 0.29 ^b^
CAS_CAN_0.5	43.79 ± 0.16 ^a^
CAS_CAN_0.5T	43.92 ± 0.28 ^ac^
CAS_CAN_1	43.41 ± 0.27 ^a^
CAS_CAN_1T	43.61 ± 0.36 ^a^
CAS_CAR_0.5	45.38 ± 0.31 ^be^
CAS_CAR_0.5T	45.42 ± 0.12 ^b^
CAS_CAR_1	44.46 ± 0.53 ^cd^
CAS_CAR_1T	44.76 ± 0.20 ^de^

Mean values ± standard deviations. Different superscripts (^a–e^) within the column indicate significant differences between samples at *p* < 0.05. CAS—sodium caseinate, CAN—candelilla wax, CAR—carnauba wax, T—Tween-80.

**Table 4:** L*, a*, b* color parameters, total color differences (ΔE), and opacity of sodium caseinate films with candelilla and carnauba wax.

Film	L*	a*	b*	ΔE	Opacity (A·mm^−1^)
Control	98.9 ± 0.03 ^f^	−0.85 ± 0.01 ^g^	3.50 ± 0.03 ^f^	1.73 ± 0.03 ^f^	2.28 ± 0.50 ^d^
CAS_CAN_0.5	97.2 ± 0.63 ^e^	−2.21 ± 0.16 ^e^	4.84 ± 0.74 ^e^	2.75 ± 0.82 ^e^	6.40 ± 1.29 ^a^
CAS_CAN_0.5T	96.1 ± 0.84 ^a^	−2.53 ± 0.23 ^be^	7.04 ± 1.56 ^ab^	5.24 ± 1.78 ^ab^	4.62 ± 0.70 ^b^
CAS_CAN_1	96.6 ± 0.37 ^ae^	−2.37 ± 0.10 ^be^	6.73 ± 0.64 ^a^	4.75 ± 0.72 ^a^	7.03 ± 0.92 ^a^
CAS_CAN_1T	95.9 ± 0.49 ^a^	−2.82 ± 0.12 ^cd^	7.48 ± 0.87 ^abc^	5.74 ± 0.99 ^abc^	5.45 ± 0.92 ^ab^
CAS_CAR_0.5	95.9 ± 0.63 ^ab^	−2.56 ± 0.14 ^be^	8.90 ± 0.01 ^c^	7.04 ± 1.35 ^c^	6.85 ± 0.96 ^a^
CAS_CAR_0.5T	95.9 ± 0.48 ^ab^	−2.62 ± 0.17 ^ad^	8.43 ± 0.74 ^bc^	6.60 ± 0.86 ^bc^	5.71 ± 0.45 ^ab^
CAS_CAR_1	95.1 ± 0.45 ^bd^	−2.95 ± 0.16 ^c^	12.7 ± 0.97 ^d^	10.9 ± 1.06 ^d^	10.55 ± 1.64 ^c^
CAS_CAR_1T	94.1 ± 0.87 ^c^	−3.22 ± 0.16 ^f^	13.8 ± 1.49 ^d^	12.3 ± 1.69 ^d^	10.05 ± 1.77 ^c^

Mean values ± standard deviations. Different superscripts (^a–g^) within any column indicate significant differences between samples at *p* < 0.05. CAS—sodium caseinate, CAN—candelilla wax, CAR—carnauba wax, T—Tween-80.

**Table 5:** Water vapour permeability (WVP) of sodium caseinate films with candelilla and carnauba wax.

Film	WVP (×10^−10^ g·m^−1^·Pa^−1^·s^−1^)
Control	4.75 ± 0.10 ^e^
CAS_CAN_0.5	3.52 ± 0.07 ^cd^
CAS_CAN_0.5T	2.83 ± 0.04 ^a^
CAS_CAN_1	3.06 ± 0.14 ^abc^
CAS_CAN_1T	2.66 ± 0.27 ^a^
CAS_CAR_0.5	3.90 ± 0.11 ^d^
CAS_CAR_0.5T	3.66 ±0.12 ^d^
CAS_CAR_1	3.45 ± 0.25 ^bcd^
CAS_CAR_1T	3.05 ± 0.18 ^ab^

Mean values ± standard deviations. Different superscripts (^a–e^) within the column indicate significant differences between samples at *p* < 0.05. CAS—sodium caseinate, CAN—candelilla wax, CAR—carnauba wax, T—Tween-80.

**Table 6:** Tensile strength (TS), Young’s modulus (YM), and elongation at break (E) of sodium caseinate films with candelilla and carnauba wax.

Film	TS (MPa)	E (%)	YM (MPa)
Control	1.65 ± 0.42 ^a^	142. 0 ± 31.3 ^ef^	0.26 ± 0.06 ^a^
CAS_CAN_0.5	2.30 ± 0.49 ^a^	120.1 ± 25.9 ^de^	0.64 ± 0.18 ^abc^
CAS_CAN_0.5T	4.78 ± 1.11 ^b^	159.9 ± 22.4 ^f^	1.66 ± 0.50 ^d^
CAS_CAN_1	2.60 ± 0.7 ^a^	68.9 ± 17.1 ^ab^	0.62 ± 0.10 ^abc^
CAS_CAN_1T	4.73 ± 1.51 ^b^	102.4 ± 16.5 ^bcd^	1.78 ± 0.55 ^d^
CAS_CAR_0.5	1.91 ± 0.34 ^a^	76.6 ± 24.2 ^abc^	0.48 ± 0.15 ^ab^
CAS_CAR_0.5T	2.50 ± 0.43 ^a^	114.5 ± 29.9 ^cde^	0.68 ± 0.15 ^abc^
CAS_CAR_1	2.28 ± 0.72 ^a^	63.7 ± 19.5 ^a^	0.71 ± 0.15 ^bc^
CAS_CAR_1T	2.84 ± 0.65 ^a^	93.4 ± 21.1 ^abcd^	0.96 ± 0.12 ^c^

Mean values ± standard deviations. Different superscripts (^a–f^) within any column indicate significant differences between samples at *p* < 0.05. CAS—sodium caseinate, CAN—candelilla wax, CAR—carnauba wax, T—Tween-80.
